# Clinically proven mtDNA mutations are not common in those with chronic fatigue syndrome

**DOI:** 10.1186/s12881-017-0387-6

**Published:** 2017-03-16

**Authors:** Elizna M. Schoeman, Francois H. Van Der Westhuizen, Elardus Erasmus, Etresia van Dyk, Charlotte V. Y. Knowles, Shereen Al-Ali, Wan-Fai Ng, Robert W. Taylor, Julia L. Newton, Joanna L. Elson

**Affiliations:** 1 Centre for Human Metabolomics, North-West University, Potchefstroom, South Africa; 2 Institute of Cellular Medicine, Faculty of Medical Sciences, Newcastle University, Newcastle upon Tyne, UK; 3 Department of Biology, College of Science, University of Basrah, Basrah, Iraq; 4 Wellcome Trust Centre for Mitochondrial Research, Institute of Neuroscience, The Medical School, Newcastle University, Newcastle upon Tyne, NE2 4HH UK; 5 Newcastle Hospitals NHS Foundation Trust, Newcastle upon Tyne, UK; 6 Institute of Genetic Medicine, Newcastle University, Central Parkway, Newcastle upon Tyne, UK

**Keywords:** Chronic fatigue syndrome, mtDNA mutations, Pathogenicity

## Abstract

**Background:**

Chronic Fatigue Syndrome (CFS) is a prevalent debilitating condition that affects approximately 250,000 people in the UK. There is growing interest in the role of mitochondrial function and mitochondrial DNA (mtDNA) variation in CFS. It is now known that fatigue is common and often severe in patients with mitochondrial disease irrespective of their age, gender or mtDNA genotype. More recently, it has been suggested that some CFS patients harbour clinically proven mtDNA mutations.

**Methods:**

MtDNA sequencing of 93 CFS patients from the United Kingdom (UK) and South Africa (RSA) was performed using an Ion Torrent Personal Genome Machine. The sequence data was examined for any evidence of clinically proven mutations, currently; more than 200 clinically proven mtDNA mutations point mutations have been identified.

**Results:**

We report the complete mtDNA sequence of 93 CFS patients from the UK and RSA, without finding evidence of clinically proven mtDNA mutations. This finding demonstrates that clinically proven mtDNA mutations are not a common element in the aetiology of disease in CFS patients. That is patients having a clinically proven mtDNA mutation and subsequently being misdiagnosed with CFS are likely to be rare.

**Conclusion:**

The work supports the assertion that CFS should not be considered to fall within the spectrum of mtDNA disease. However, the current study cannot exclude a role for nuclear genes with a mitochondrial function, nor a role of mtDNA population variants in susceptibility to disease. This study highlights the need for more to be done to understand the pathophysiology of CFS.

**Electronic supplementary material:**

The online version of this article (doi:10.1186/s12881-017-0387-6) contains supplementary material, which is available to authorized users.

## Background

CFS is a chronic debilitating condition affecting approximately 600,000 people in the UK alone [1]. Fatigue is the defining clinical problem, it is a fatigue that is not eliminated by sleep or rest. Differences in exercise recovery in those with CFS have been demonstrated, with patients showing a reduced capacity to recover from acidosis on repeat exercise [2]. Other studies in those with CFS showed additional features suggesting a specific exercise-related defect [3]. Together these and other studies are suggestive that mitochondrial dysfunction plays a role in the pathophysiology of CFS. Additionally, a number of publications have suggested that mtDNA variation is important in the course of CFS [4, 5]. Moreover, it has been suggested that the “symptoms of mitochondrial diseases and CFS frequently overlap and can easily be mistaken”, and thus it is conceivable that some CFS patients may harbour clinically proven mtDNA mutations [6].

Clinically proven mtDNA mutations are an important cause of inherited neuromuscular disease, frequently presenting as multisystem disorders with fatigue being prevalent in patients with mitochondrial disease [7, 8]. Most pathogenic mtDNA mutations are heteroplasmic, that is wild-type and mutated mtDNA co-exist in the cells and tissues of affected patients. Typically the mutant allele would have to be present at 60% or greater for a biochemical defect to be observed, termed the “threshold effect” [9]. The expression of different clinical phenotypes in patients is presumed in part to relate to heteroplasmy levels within affected tissues. The threshold for a phenotype depends on the specific mtDNA mutation, and other factors that typically modify the expression of a pathogenic variant [9]. It is unknown if clinically proven mtDNA mutations at levels insufficient to cause clinically manifesting mitochondrial disease, could be a risk factor for a particular complex disease, or alter the course of disease after the person has been affected.

To investigate the possibility of CFS patients harbouring clinically proven mtDNA mutations, either above the threshold required for mtDNA disease or at a sub-threshold level, the complete mitochondrial genome of 93 CFS patients, was sequenced using next generation sequencing as described [10]. We used two well-characterised cohorts of CFS patients, one form the North East of England (*n* = 52) and the other from the North-West Province of South Africa (*n* = 41). The CFS patients (aged 42.94 years s.d +/- 14.6, *n* = 93) in both cohorts were identified following conventional clinical screening approaches and met the Fukuda diagnostic criteria [11]. Potentially confounding causes of fatigue, including depression, were excluded in all patients.

## Methods

Total DNA was isolated from blood samples using QIAamp DNA Blood Mini Kits (Qiagen). Massively parallel DNA sequencing of the PCR fragments were performed in both strands using an Ion Personal Genome Machine® (PGM™) Sequencer. Libraries were prepared using Ion Plus Fragment library kits (LifeTechnologies). Ion Xpress™ Barcode Adapters were used instead of the universal adaptors during library preparation. Primary data analysis was performed using the CLC Genomics Workbench 4.6.1 (CLC bio). Standard Flowgram Format files were imported and trimmed in order to remove low quality nucleotides, using default settings. High quality sequencing reads for each patient were mapped against the revised Cambridge Reference Sequence (rCRS) of human mtDNA (GenBank NC_012920), using default settings, in order to obtain a consensus sequence for each individual and to enable variation detection. Single nucleotide polymorphisms (SNPs) were automatically detected using the high-throughput sequence SNP detection function, which also enables the estimation of variant allele frequency (%), as performed in van der Walt EM et al. [10].

## Results

The variants seen in each of the 93 CFS patients are listed in Additional file 1: Table S1, the lists were compiled by comparison of consensus sequence to the standard reference sequence the revised Cambridge Reference Sequence (rCRS). We compared mtDNA variants seen in the CFS patients (Additional file 1: Table S1) to a list of mtDNA variants reported as pathogenic or associated with disease on the MitoMap database [12, 13]. Variants on the list of disease associated variants also seen in the CFS cohort were evaluated by reference to the reporting literature and applying accepted criteria to evaluate the evidence for pathogenicity presented in the reporting literature [14] to ensure the variants could be classified as proven mtDNA mutations.

The average coverage was 398 reads, with heteroplasmies of as low as 10% being considered in preliminary analyses when these positions met certain criteria [10], including to be near the average coverage. Using the sequnce data, we detected three clinically-validated mtDNA mutations in the CFS patients substantially below the levels required to cause mtDNA disease these were m.7497G > A [15, 16], m.9185 T > C [17] and m.10197G > A [18], see Additional file 1: Table S1. The low frequency clinically-proven variants detected in the first phase of the analysis appeared to be genuine low-level heteroplasmies. Given clinically-proven mtDNA variants at low frequencies below that required to cause mtDNA disease were of particular interest in this study, it was important to carefully consider such findings and conduct rigorous validation. Upon further investigation of these low frequency mtDNA variants using pyrosequencing, which provides the diagnostic gold standard for quantification of mtDNA heteroplasmy, the presence of these variants was not confirmed and they were thus considered as artefacts of the initial NGS on the Ion Torrent.

## Discussion

This study did not find evidence that any of our 93 CFS patients diagnosed with the Fukuda criteria were suffering from undiagnosed mtDNA disease, nor did it uncover low levels of clinically proven mtDNA mutations that might be a susceptibility factor for CFS, or impacting on the course of disease. Initial exploratory sequencing using NGS on the Ion Torrent indicated this was potentially the case, but subsequent investigation applying pyrosequencing did not support this assertion. This demonstrates the need to validate such observations on platforms accepted for quantification in the diagnostic context. Blood is still used in diagnostic practice, and was used in this study. However, it is known that the heteroplasmy levels of mtDNA mutations can vary between tissues, with some mutations in particular the m.3243A > G mutation being known to decline in blood with age [19, 20]. It has been shown that for the m.3243A > G mutation urinary epithelium is a more reliable indicator of the level of mutations in skeletal muscle [21]. Despite this, in the absence of evidence of the presence of any clinically proven mtDNA mutations in the 93 patients studied here, the collection of additional tissues for study would be difficult to justify, especially the collection of skeletal muscle, which would entail an invasive muscle biopsy. Deeper sequencing or targeted mutation detection would be capable of detecting lower levels of mtDNA mutation than we could detect in this study. A study using a targeted detection method has been used previously to show as many as 1/200 healthy individuals carry very low levels of a known mtDNA mutation [22]. However, given the high levels of heteroplasmy required to see a biochemical defect in mitochondrial patients [9] it becomes biologically less plausible that very low levels of mtDNA mutations would be exerting a phenotypic effect even in the context of co-occurrence with a disease phenotype such as CFS. The data supports the view that CFS does not fall within the spectrum of inherited mtDNA disorders, as there is an absence of clinically proven mutations at appreciable levels of heteroplasmy in the 93 patients sequenced for this study.

This work does not exclude a role for mtDNA population variation in the susceptibility to CFS as such, mtDNA variation has been linked to other complex diseases, including multiple sclerosis [23] where fatigue is part of the clinical presentation. In investigating the possibility that mtDNA population variation might play a role in susceptibility to CFS, or impact on the course of disease, alternative models such as the mutational load hypothesis should be considered [24] as well as the traditional haplogroup association model, as the later model has been the subject of increasing levels of critical comment [25]. However, to make the breakthough in understanding the genetics of this complex condition a large scale-sequencing project will need to be undertaken to help discover the genes and pathways that differ in those suffering from this complex condition. Data from such a “big data” genomics project will help us understand how this disease is stratified, which in turn should help to guide the way that patients are treated, as well as providing therapeutic targets.

## Conclusions

The work supports the view that CFS should not be considered to fall within the spectrum of disease caused by or associated with clinically proven mtDNA mutations. That is CFS patients with mtDNA mutations are likely to be rare. This has implications for suggested treatment modalities of CFS patients, and for the suggested clinical follow-up of CFS patients.

## Additional file

Additional file 1: Table S1. Contains the mtDNA variants from participants. (XLSX 24 kb)

## Abbreviations

CFS: Chronic fatigue syndrome
MtDNA: Mitochondrial DNA
rCRS: Cambridge reference sequence
RSA: South Africa
UK: United Kingdom
